# The influence of predation risk on mate signaling and mate choice in the lesser waxmoth *Achroia grisella*

**DOI:** 10.1038/s41598-020-57481-1

**Published:** 2020-01-16

**Authors:** Christopher Edomwande, Flavia Barbosa

**Affiliations:** Lake Forest College, Department of Biology, Lake Forest, IL 60045 USA

**Keywords:** Ecology, Evolution

## Abstract

Mating signals are often conspicuous and can be eavesdropped on by predators. Therefore, it is expected that predation risk will shape the evolution of sexual communication in both senders and receivers. Males of the lesser waxmoth *Achroia grisella* produce ultrasonic signals to attract females, and females have preferences for certain signal traits. When they detect predator cues such as bat echolocation calls, males typically stop signaling and females freeze. Here we examine different ways in which predation risk affects male signaling and female choice. We exposed calling males to predator cues under three different treatments: in isolation, within a simulated lek composed of attractive males, or within a simulated lek of unattractive males. We also tested if female choice depends on predation risk by submitting females to a two-choice test between an attractive and an unattractive signal, in the presence and the absence of predator cues. We found that risk-taking behavior is not only impacted by the presence of competitors, but by the competitors’ attractiveness as well. Additionally, more attractive males take more risks when calling in isolation, but not when lekking. In females, we found that preference for the more attractive call disappeared when the attractive call was associated with higher predation risk. These results reinforce the trade-off between traits that increase survival and mate attraction, and bring new insight into the evolution of leks in this species.

## Introduction

Mating is a crucial activity in the life history of sexually reproducing animals, and most of the time, it involves conspicuous behaviors and structures^1–3^. In many species, males possess ornaments, perform elaborate courtship or broadcast sexual signals to attract females. These conspicuous traits, however, can also attract the attention of predators^4–6^. In fact, the signals that are most attractive to females are often the most conspicuous ones, making attractive males even more likely to be eavesdropped on by predators^7^. As a result, signalers must consider this trade-off between attracting mates and exposing themselves to predation risk^3,5^.

Attracting mates has long been recognized as a hazardous activity for males, but behaviors involved in mate choice can also put females under higher predation risk. As females move around breeding sites sampling different males and choosing mates, they are also vulnerable to predators attracted to the area by those males’ signals and by cues resulting from their own movement^8,9^. Additionally, females often become more vulnerable to predators during copulation, being overall under similar or even higher predation risk than males^10–12^. As a result, both sexes often change their behavior under different levels of predation risk, so to balance out the opposing forces of natural and sexual selection. For example, in the presence of predators, males may decrease signal intensity or even stop signaling altogether^13–16^. Meanwhile, females in the process of evaluating males may arrest movement to decrease their conspicuousness^14,17^, or they may change their choice behavior by becoming less discriminating and at times selecting mates that do not align with their underlying signal preferences^18–21^.

The interplay between finding mates and avoiding predators becomes more complex when we account for other factors that may increase the relative value of mating or the risk level for an individual. For example, the cost of missed mating opportunities is generally higher for males than it is for females, leading to the prediction that males should take more risks when engaging in reproductive behaviors^8,22–24^. Missed opportunity costs also depend on the age of the individuals, and both sexes are expected to invest more in terminal mating opportunities and take more risks as they age^25–27^. Finally, the social environment also plays a role in the relative costs and benefits of seeking mates, introducing the potential of further behavioral adjustments in response to eavesdroppers based on the number and identity of competitors present in the breeding site^28–30^. This can be observed particularly well in lekking and chorusing species, where both males and females have the opportunity to adjust their behavior based on the individual traits of the aggregated males^31,32^.

Both males and females of the lesser waxmoth *Achroia grisella* (Pyralidae: Galleriinae) must cope with eavesdropping predators when engaging in various mating behaviors. Males aggregate in small leks at night to broadcast ultrasonic mating signals^33^. Lekking aggregations typically range from 2 to 10 males, and a lek size of 4–6 males has been shown to be optimal for per capita mate attraction^34^. Male signals consist of an incessant pulse pair train typically produced at frequencies between 70 and 110 kHz^35^. Males also produce a chemical signal, an odor secreted by glands in the forewings. This male odor plays a role in long-distance female attraction, but its role in female choice within the lek is unclear^33^. Receptive adult females come to the lekking areas to evaluate male songs and choose a mate, usually based on three call traits: pulse pair rate (PPR), peak call amplitude and interpulse interval asynchrony^36^ Once a female makes a choice, she orients herself and walks towards the male for mating^36^. Both male songs and female movements make individuals conspicuous to predators, especially echolocating bats. In turn, both sexes have been shown to modify their behavior in the presence of bat echolocation calls^37^.

When a calling lesser waxmoth male detects predator cues, he usually displays a silence response, a brief pause that can range from hundreds of milliseconds to a few minutes^38–40^. Male lifetime fitness depends on total time spent calling as waxmoths have a short adult lifespan of 7 to 14 days; therefore, this silence response comes at a cost for males. On the other hand, forgoing a silence response exposes males to higher predation risk^32,38^. There is substantial variation in the occurrence and duration of the silence response, both between and within males, and several factors influence male responses. For example, the social environment at breeding sites plays an important role: typically, isolated males produce more and longer silence responses than those calling in a lek when exposed to the same predator cues^32,34^. Another critical factor is the attractiveness of an individual’s signals, and surprisingly, attractive males take more risks when calling in isolation, producing fewer and shorter silence responses^39^. This is contrary to what is observed in other species^41–43^, and to the theoretical predictions of the “asset protection principle”, which states that individuals of lower potential reproductive value are the ones who should incur more risks^44^. The interplay between male attractiveness and risk-taking in the lesser waxmoth remains to be further explored.

In this study, we aimed to explore how the presence of predator cues affect the behavior of male and female waxmoths. In males, we investigated how risk-taking behavior is impacted by the presence of competitors, by the attractiveness of the competitors, and by the attractiveness of the individual male, in addition to the interactions between these different factors. We exposed calling males to predator cues under three different social environment treatments: in isolation, within a simulated lek composed of attractive males, and within a simulated lek composed of unattractive males. We hypothesized that male risk-taking behavior would depend not only on the presence of competitors, but on the attractiveness of the competitors as well. Therefore, we predicted that males would exhibit fewer and shorter silence responses when calling within a lek of more attractive males. We also predicted that more attractive males would exhibit fewer and shorter silence responses under all treatments. A previous study has demonstrated that attractive males take more risks in this species, but they only investigate males calling in isolation. In females, we investigated if mate choice depends on predation risk. We gave receptive females a choice between an attractive and an unattractive male signal, with and without a bat call being broadcast from the same direction as the more attractive call. We predicted that females would be more likely to choose the attractive call in the absence of bat cues, but that this preference would disappear when the predation cue was broadcast.

## Methods

### General setup

Our study individuals come from a laboratory population in Lake Forest College, Lake Forest, Illinois, USA that was started from a few hundred individuals collected from a wild population near Woodsville, Mississippi, on October 2017. The laboratory population is housed and maintained at 25 ± 1 °C under a 12:12 L:D photoperiod and fed an artificial diet consisting of flour, honey, glycerin, beeswax, and nutritional yeast (see^36^ for details on diet). Under these conditions, larvae take between 3 and 4 weeks to pupate, and pupae take 7 to 10 days to emerge as adults^27^. Adults do not feed and typically live for 10 to 14 days^33^. They become sexually receptive shortly after emergence, and males promptly start calling. Males mate multiple times, but females typically only mate once^45,46^. Therefore, to standardize physiological state and sexual receptivity for study individuals, we conducted all tests with unmated adults between 1 and 3 days of age post-emergence. We collected pupae from the general population and isolated them in plastic cups of approximately 30 cm^3^. We checked isolated pupae daily for emergences, and adults were promptly sexed and massed on a microbalance (Mettler-Toledo XP26).

**Figure 1:**
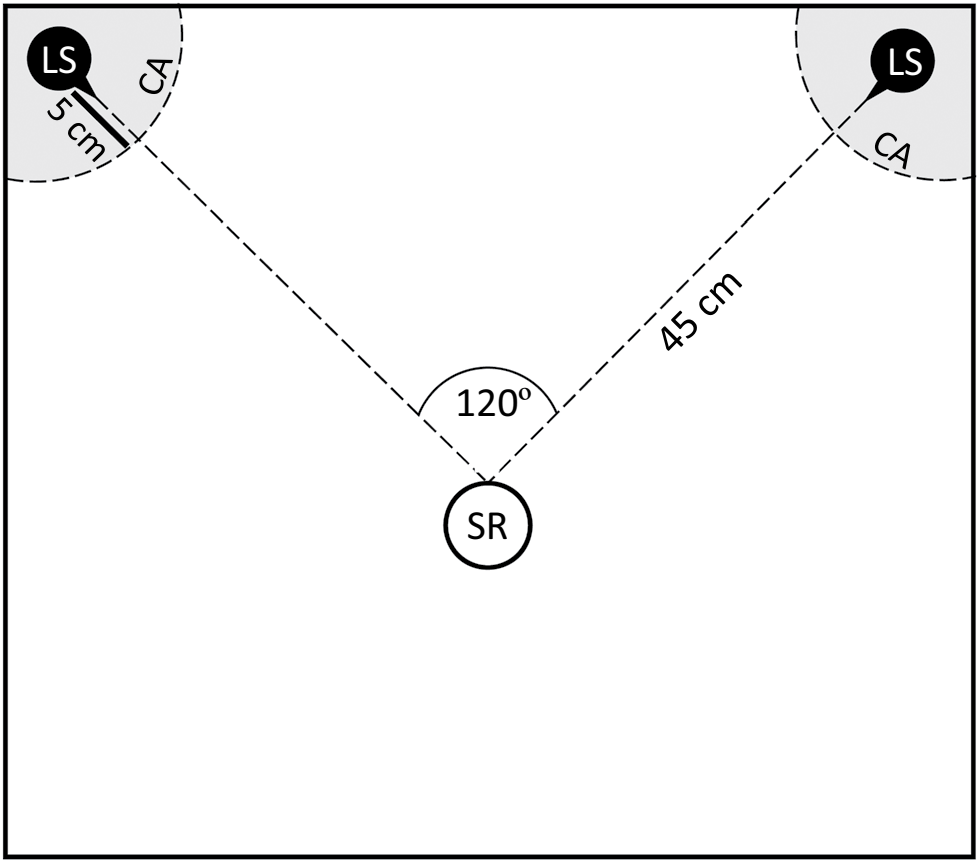
Testing arena. The starting region (SR) was 45 cm away from two loudspeakers (LS) in adjacent corners of a custom-built wood and cardboard arena. Choice area (CA) was designated by 5 cm diameter semicircles around each loudspeaker for phonotaxis tests.

All tests were conducted between March and October 2018, at 25 ± 1 °C, during the first four hours of the night, which is the time when individuals are most active. The testing room was kept dark, and behaviors were observed under diffuse light from a 25 W incandescent red bulb. We conducted tests on a 60 × 60 cm custom-built wood and cardboard arena surrounded by an acoustically-isolating foam barrier. We placed loudspeakers (ScanSpeak, AviSoft Bioacoustics, frequency range 60–110 kHz) in two adjacent corners, 45 cm away from the starting region, a 5 cm diameter circle where tests individuals were placed for trials. The loudspeakers face the starting region (SR) forming a 120 °C angle (see Fig. 1). For female choice tests, we also demarcated 5 cm diameter semicircles around the loudspeakers and designated those the choice area (CA).

### Experiment 1 - Male risk-taking behavior

We recorded calls of 26 unmated, adult males before and after exposure to a predator cue under each of three social environment treatments: male in isolation, male in a simulated lek with attractive competitors, or male in a simulated lek with unattractive competitors. We randomized the order in which each male was submitted to the three treatments and waited for a minimum of 30 minutes before consecutive trials to prevent the focal individual from becoming habituated to test signals. Before and between tests, males were housed individually in plastic cups which were placed within a grid of acoustic insulating foam barriers to prevent them from being exposed to any other acoustic cues. At the onset of tests, males were placed in acoustically transparent mesh cages.

The predator cue we used was a synthetic bat echolocation call created according to the methods and parameters in^27^. Briefly, it represented the search phase call of a gleaning bat, which would be a likely threat to an insect species that moves and calls from a substrate such as the lesser waxmoth^37^. The synthetic call we used consists of a 2.5 ms pulse with a downward frequency sweep descending from 90 kHz to 30 kHz over its length. This pulse was then used to produce a train of 20 pulses per second. During tests, the synthetic bat call was broadcast from a third, overhead speaker, not represented in Fig. 1.

The simulated leks within which focal males called consisted of acoustic stimuli broadcast from the two loudspeakers on adjacent corners of the testing arena. To create the stimuli used in experiments, we selected a single pulse pair from a previous recording of this same population of moths to create a continuous train of pulse pairs using the software AviSoft SASLab Pro (AviSoft). We chose a pulse pair whose temporal and spectral properties were typical of the population. We created two stimuli differing in pulse pair rate (PPR): one at 65 pulse pairs s^−1^ and another at 95 pulse pairs s^−1^. Both these values for PPR fall within the range of this population, but females prefer higher PPR^39,47^. Therefore, the rate of 65 pulse pairs s^−1^ represents an unattractive call, and 95 pulse pairs s^−1^ represents an attractive call. PPR is one of three call traits females have a preference for in this species, along with amplitude and pulse pair gap^36^. We chose to focus on PPR because call amplitude also depends on the distance between signalers, and we did not want to introduce this potential confounding factor. In addition, female preference based on pulse gap width has been demonstrated to be significantly weaker than that for PPR^36^. We kept all other signal traits identical between the two call stimuli, including amplitude and pulse pair gap. Our three social environment treatments, therefore, correspond to the following: isolation, where nothing was broadcast from the two loudspeakers; simulated lek with unattractive competitors, where two identical calls of 65 pulse pairs s^−1^ were broadcast, and simulated lek with attractive competitors, where two identical calls of 95 pulse pairs s^−1^ were broadcast. From here on we will refer to these treatments as silence, low PPR lek and high PPR lek respectively. We should point out that the male calls used to simulate a lek do not include silence responses, as live males would. However, a previous study compared the responses of males to bat stimuli when calling with an artificial lek simulated by recordings versus a natural lek of live males and found no overall differences on how males respond^32^.

We began each trial by placing a focal male in a mesh cage and placing the cage in the SR region of the arena. The focal male was allowed to acclimate for 5 minutes, and during this time the loudspeakers played either silence or one of the two simulated lek treatments in a continuous loop until the end of the trial. For playback, we used the software AviSoft Recorder (AviSoft Bioacoustics) on a laptop connected to ultrasonic loudspeakers (ScanSpeak, AviSoft Bioacoustics, frequency range 60–110 kHz) and a four-channel amplifier (UltraSoundgate Player 416 H, AviSoft Bioacoustics). We adjusted playback peak amplitude to 75 dB SPL using a sound pressure level meter and the peak equivalence method^27^. After the acclimation period, we recorded the focal male for one minute. If the focal male were not singing within five minutes, we would start a new test with a different male and re-test the previous male at a different time, after a minimum of 30 minutes. After this baseline recording, we played the bat cue from the overhead speaker for 1 second, and we continued recording the focal male for 3 additional minutes, starting at the same time as the bat cue playback. For recordings, we used a condenser ultrasound microphone (CM16/CMPA, AviSoft Bioacoustics, frequency range: 2 kHz − 200 kHz) along with an analog/digital converter (UltraSoundGate 416 H, AviSoft Bioacoustics) and the AviSoft RECORDER USGH software (AviSoft Bioacoustics), which recorded the file at 16 bits and 500,000 samples s^−1^. We tested a total of 26 males through the 3 treatments.

From our recordings, we measured the focal male’s PPR before and after the bat playback. We also scored recordings for the presence or absence of a silence response post bat playback and measured the silence response duration when it was present. We used the software AviSoft SASLab Pro to visualize and measure our sound files.

### Experiment 2 - Female choice under predation risk

We tested female response to predator cues through a two-choice phonotaxis test. Each focal female was tested twice, under the presence and absence of a predator cue. In each test, she was given a choice between a less and a more attractive male calls (65 and 95 pulse pairs s^−1^ respectively). In one of the tests, we added a bat call which was broadcast from a third speaker, situated in the same direction but above the higher PPR male call. The stimuli and playback methods were the same as described above for male tests. We tested a total of 18 females under both conditions, and we randomized among them the testing order they would go through, as well as the position of the male calls and bat cue in the arena (left or right). As with males, females were individually placed in a plastic cup within a grid of acoustic insulating foam barriers before and between tests and were given a minimum of 30 minutes between tests to prevent habituation.

Prior to testing, females were placed under an acoustically transparent mesh funnel on the SR region of the arena for a 5-minute acclimation period. We started the playback of the male stimuli at the beginning of the acclimation period and after 5 minutes, we either played silence or 1 s bat call from the third speaker. We lifted the mesh funnel, allowing the female to move around the arena, and we recorded the female movement in the arena and scored choice of a stimulus when she reached the choice area (CA) near the speaker. The male calls continued to play in a loop until the end of the test. We ended the test either when the female made a choice, or when she failed to make a choice for 15 minutes. Females that did not make a choice in the absence of the bat call were considered unresponsive and were not included in the analysis. We tested a total of 18 responsive females in two trials.

### Statistical analysis

We performed all statistical analysis with JMP Software (Version 13, SAS Institute Inc., Cary, NC). For experiment 1, we tested if the number of times males exhibited a silence response varied among our treatments using the Cochran Q test and McNemar tests for posthoc pairwise comparisons with Bonferroni corrections to check for differences among groups. Then, we tested if the length of silence responses differed among treatment groups using a One-Way ANOVA with repeated measures, followed by Tukey HSD for posthoc pairwise comparisons. Finally, we tested for the effect of the focal male’s own attractiveness, treatment, and the interaction between them on silence response duration using a univariate SLS model where male ID was included as a random effect to account for our repeated measures design. For experiment 2, we tested if females have a preference for the attractive signal in each of the two treatments with Chi-Square tests of goodness-of fit. We then tested if treatment had an effect on female preference for the most attractive call with a McNemar test.

## Results

### Experiment 1 - Male risk-taking behavior

**Figure 2:**
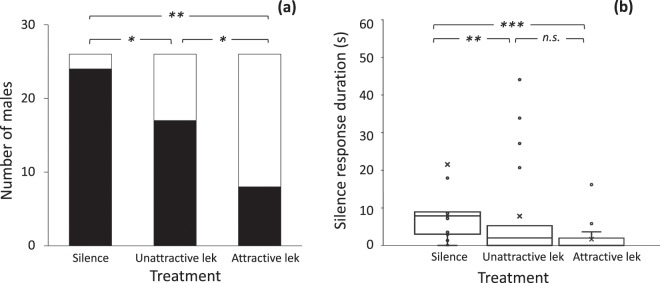
The likelihood and duration of the male silence response varied with experimental treatment. (**a**) Males exhibited the silence response (black) more frequently when calling in isolation, while calling continuously (white) more often in the attractive lek treatment. Asterisks indicate statistically significant differences in pairwise comparison at an alpha level of 0.016 (*p < 0.016, **p < 0.001). (**b**) Silence responses were longer when males were calling in isolation. Box plots show mean (line within box), upper and lower quartiles (box), range (whisker) and outliers (circles). Asterisks indicate statistically significant differences in pairwise comparison at an alpha level of 0.05 (**p < 0.01, **p < 0.001).

We found an overall effect of treatment on the likelihood of males exhibiting a silence response (Cochran Q test: X^2^ = 23.21, p < 0.0001, n = 26, Fig. 2a). Males were more likely to exhibit a silence response when calling in isolation, followed by when in a low PPR simulated lek, and had the highest risk-taking responses when in a high PPR simulated lek. Posthoc tests revealed statistically significant differences between all three pairwise comparisons at an alpha level of 0.016 post Bonferroni corrections (McNemar test, silence vs. unattractive: X^2^ = 6, p = 0.015; silence vs. attractive: X^2^ = 11, p < 0.001; unattractive vs. attractive: X^2^ = 6.5, p = 0.012, Fig. 2a).

We also found that the duration of male silence response varied with treatment (One-Way ANOVA with repeated measures, F_2,25_ = 12.88, p < 0.0001, Fig. 2b). Males exhibited longer silence responses when calling in isolation, but there was no difference in silence response length between low and high PPR simulated lek treatments (Tukey HSD, silence vs. unattractive p < 0.0005, silence vs. attractive p < 0.0001, unattractive vs. attractive p = 0.71, Fig. 2b).

**Figure 3:**
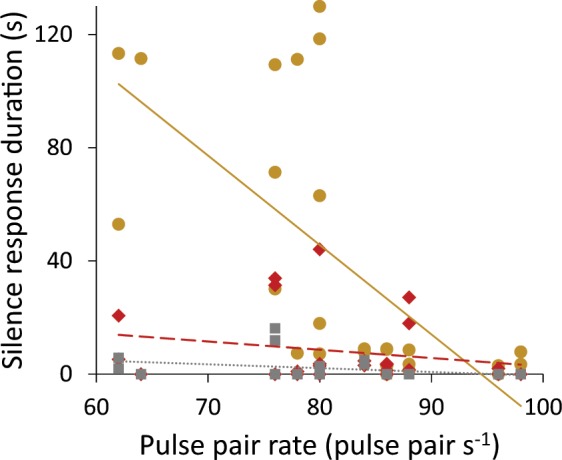
There is an interaction effect between treatment and focal male attractiveness in male silence response duration. More attractive males took more risks by having shorter silence responses, but only when calling in isolation (gold round markers, solid line). Males calling within an unattractive lek (red diamond markers, dashed line) and within an attractive lek (gray square markers, dotted line) had shorter silence responses overall regardless of their own call attractiveness.

Finally, we found that the duration of the silence response was significantly affected by treatment, focal male attractiveness, and the interaction between treatment and focal male attractiveness. We measured focal male attractiveness of his baseline call PPR (SLS Univariate Model, treatment: F_2,51_ = 20.66, p < 0.001; attractiveness: F_1,51_ = 20.54, p < 0.001; treatment*attractiveness: F_2,51_ = 13.88, p < 0.001). Specifically, more attractive males took more risks by producing shorter silence responses when calling in isolation. However, when calling in leks, males had shorter silence responses overall, regardless of their own attractiveness (Fig. 3).

### Experiment 2 - Female choice under predation risk

**Figure 4:**
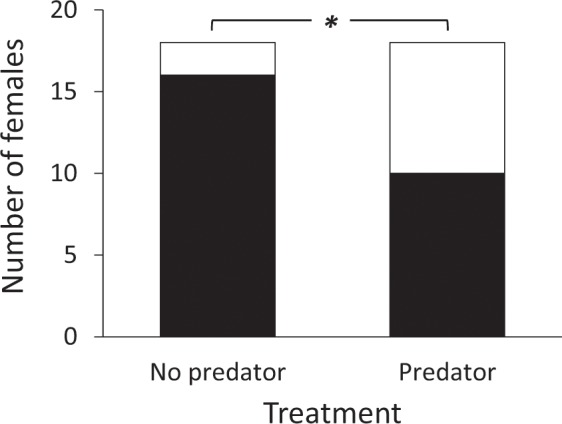
Female preference for the higher PPR call disappeared under the presence of a predator cue. Figure shows the number of females that chose the higher PPR call (black) and the lower PPR call (white) under two experimental treatments. Asterisk indicates statistically significant difference at an alpha level of 0.05.

Females, as expected, had a preference for the higher PPR calls in the absence of predator cues (χ^2^ = 10.89, p < 0.001). However, when the predator call was broadcast from the same direction as the more attractive call, this preference disappeared (χ^2^ = 0.22, p = 0.64). The proportion of females that preferred the attractive call varied between the two treatments (McNemar test, χ^2^ = 5.25, p = 0.02, Fig. 4).

## Discussion

Lesser waxmoth males and females adjust their mating behavior in response to predation risk. Most notably, male response to predator cues is context-specific in that their response varies not only with male identity, but with the relative costs and benefits of risk-taking behavior under different social environments. Furthermore, even though females have underlying preferences for particular male call traits, we found that their choice of mate is affected by the risk level of the environment, which may lead to a mismatch between choice and preference. This general pattern has been previously documented in numerous species, demonstrating how individuals balance selection for traits that increase survival versus mate attraction and mate choice^1^.

We found that solitary males take fewer risks than those calling within a lek, as previously reported^32,34,40^, by exhibiting more and longer silence responses. In addition, we demonstrated that the overall attractiveness of surrounding male competitors plays a role on risk-taking response as well. For example, when surrounded by a more attractive lek, males were less likely to exhibit a silence response than when surrounded by a less attractive lek. This pattern may be explained by several, non-mutually exclusive hypotheses: first, predation risk may be lower in leks due to a predator dilution effect^48^. Second, competition for mates may make the cost of a silence response higher for lekking males. Finally, the predator signal may be masked by the song of neighboring males. A previous study has demonstrated that both the predator dilution effect and signal masking operate in lesser waxmoth leks, as the per capita predation risk is indeed lower in larger groups in this species^34^. Here we demonstrate that the effect of male-male competition also plays a major role in male risk-taking behavior: males take more risks based not only on the presence of competitors but on the competitor attractiveness as well. This makes sense as mate competition should intensify when opponents are more attractive.

We also found that there is an interaction between a male’s own attractiveness and his social environment while signaling. More attractive males take more risks by exhibiting shorter silence responses when calling in isolation^39^. However, we found that this relationship disappears when competitors are present: in a lek, males produce overall short silence responses that are not affected by the individual’s own attractiveness. A previous work that demonstrated that attractive males take more risks also examined the effect of the social environment in risk-taking behavior. In that case, the authors focused on resource competition during the larval stage and did not find an effect of that social environment on risk-taking response^39^. Here we examined the effect of competition at the breeding site, which is likely to exert much stronger pressure on signaling adult males by increasing the relative cost of exhibiting a silence response.

Although it is clear that risk-taking behavior in male waxmoths depends both on the individual’s own attractiveness and the attractiveness of his competitors within a lek, it is not known if attractiveness affects a male’s conspicuousness to bats. In other species, male attractiveness at times plays a role in how conspicuous they are to eavesdroppers. For example, in tungara frogs, male mating calls are eavesdropped by predators and parasites, but the effect of male attractiveness varies for different eavesdropping species. More attractive mating calls are easier to localize by one of their predators, the frog-eating bat, resulting in higher predation risk for males broadcasting complex calls which are preferred by females^49^. On the other hand, the blood-sucking flies that are also attracted by tungara male signals do not discriminate between calls of different attractiveness^50^. In waxmoths, a previous study tested whether bats have a preference for different sized leks, and found that increased lek size did not increase conspicuousness to the predator^40^. However, it is not known whether lek attractiveness affects conspicuousness to predators in this species.

From the female perspective, predation risk also leads to changes in behavior while searching for mates. We confirmed that female preference is based on call pulse pair rate, as the majority of females we tested chose the highest pulse-pair rate call in a two-choice phonotaxis test. However, this preference disappeared when a predator cue was broadcast from the same direction as the most attractive call. Even though females do not produce acoustic cues that could be eavesdropped on by predators, their movement towards males can potentially alert predators. Thus, they are vulnerable to bats that are attracted by the calls of the female’s chosen mate. Therefore, it is reasonable that several females in our experiments chose the call that is not associated with predation risk. Surprisingly, all of the females we tested chose a male call when exposed to a bat call, rather than showing a movement arresting response for the remainder of the test. However, we should note that females also have a short adult lifespan of up to 10 days, which may contribute to their motivation to mate within a short timeframe.

We have demonstrated that a large proportion of females choose males with less preferred calls under predation risk, but we do not know whether mating with these less attractive males is costly for females. Although males mate multiply, female lesser waxmoths typically mate only once in their lifetime, with only 10 to 15% of females accepting a second mate in the laboratory^36^. This exacerbates whatever potential costs mating with less attractive males may have. Remating tends to occur when a female’s first mate is sperm depleted^46^. However, whether or not they would more likely remate after mating with a less attractive male remains an open question.

In conclusion, our findings have important implications for the understanding of the interplay between the opposing forces of natural and sexual selection. We demonstrated that both males and females make behavioral adjustments to account for predation risk while searching for mates, and we highlight the role of the social environment in determining the costs and benefits of different strategies.

## Data Availability

The datasets generated during and/or analyzed during the current study will be available in the Dryad depository upon publication.
